# *CpG *methylation of the *FHIT*, *FANCF*, *cyclin*-*D2*, *BRCA2 and RUNX3 genes *in Granulosa cell tumors (*GCTs*) of ovarian origin

**DOI:** 10.1186/1476-4598-3-33

**Published:** 2004-12-01

**Authors:** Varinderpal S Dhillon, Mohd Shahid, Syed Akhtar Husain

**Affiliations:** 1 CSIRO Health Sciences and Nutrition, Gate No 13, Kintore Avenue, PO Box 10041, Adelaide BC, Adelaide SA 5000, Australia; 2 Human Genetics Laboratory, Department of Biosciences, Jamia Millia Islamia, New Delhi, 100 025, India

## Abstract

**Background:**

Granulosa cell tumors (GCTs) are relatively rare and are subtypes of the sex-cord stromal neoplasms. Methylation induced silencing in the promoters of genes such as tumor suppressor genes, DNA repair genes and pro-apoptotic genes is recognised as a critical factor in cancer development.

**Methods:**

We examined the role of promoter hypermethylation, an epigenetic alteration that is associated with the silencing tumor suppressor genes in human cancer, by studying 5 gene promoters in 25 GCTs cases by methylation specific PCR and RT-PCR. In addition, the compatible tissues (normal tissues distant from lesion) from three non-astrocytoma patients were also included as the control.

**Results:**

Frequencies of methylation in GCTs were 7/25 (28 % for *FHIT*), 6/25 (24% for *FNACF*), 3/25 (12% for *Cyclin D2*), 1/25 (4% for BRCA2) and 14/25 (56%) in RUNX3 genes. Correlation of promoter methylation with clinical characteristics and other genetic changes revealed that overall promoter methylation was higher in more advanced stage of the disease. Promoter methylation was associated with gene silencing in GCT cell lines. Treatment with methylation or histone deacetylation-inhibiting agents resulted in profound reactivation of gene expression.

**Conclusions:**

These results may have implications in better understanding the underlying epigenetic mechanisms in GCT development, provide prognostic indicators, and identify important gene targets for treatment.

## Background

Ovarian cancer is one of the most common cancers in women of all age groups. Among ovarian neoplasms, granulosa cell tumors (GCTs) are relatively rare, accounting for approximately 3% of all ovarian cancers. One common DNA modification is promoter hypermethylation associated with loss of expression of a tumor suppressor gene. During cancer development, there is a shift in methylation patterns and some promoter region CpG islands become methylated leading to silencing of the adjacent genes [1] and this process is considered to be a critical step in cancer development. The causes of methylation change in normal cells remain unknown. It has been hypothesised that fixation of methylation occurs when random seeding of methylation occurs in the promoter regions of silent genes [2]. Methylation seems to provide an ideal set of cancer specific markers for early detection of or for monitoring response to treatment. However, the use of methylation at a given site as a marker to detect low numbers of tumor cells relies on the background levels of methylation in normal tissues to be nearly zero.

The human *FHIT *gene is a member of the histidine triad gene family [3], the function of which remains unknown. *FHIT *gene has been shown to be hypermethylated in oesophageal, lung, breast, prostate, bladder, cervical, and oral cancers [4-9]. Recent studies indicate that FANC proteins interact with both BRCA1 and BRCA2 genes through a common pathway [10]. Methylation changes may disrupt the FANC-BRCA pathway and hence may be a marker change in the cancer development in granulosa cells of the ovary. Aberrant expression of *cyclin D2 *was also demonstrated in human ovarian granulosa cell tumors and testicular germ cell tumor cell lines [11]. In breast cancer, repression of expression was attributed to methylation of the *cyclin D2 *gene promoter region. Several studies have indicated that methylation of *cyclin D2 *and its mRNA and protein were absent in most breast cancer cell lines examined and in primary breast cancers although normal breast epithelial cells had abundant expression [12-14]. BRCA2 may play role in regulation of the cell cycle during proliferation and differentiation. To date, only one study has shown the absence of methylation in the promoter region of *BRCA2 *in breast cancers cell lines and other normal human breast, bladder, colon, and liver tissues [15]. RUNX3 is one of the genes with RUNT domain, which has been identified to have a tumor suppressor role that frequently shows loss of expression due to hemizygous deletion and hypermethylation in gastric cancers [16,17].

The role of epigenetic gene inactivation in GCTs of ovarian origin is yet not fully understood. Previously published reports on GCTs and its precursor lesions showed varying degree of promoter methylation of many tumour suppressor genes [18-20]. To investigate the role of promoter methylation in detail in ovarian tumorigenesis, we further evaluated CpG methylation of 5 more tumour suppressor genes in 25 GCTs and cell lines. We found 68% of GCTs patients exhibiting promoter methylation in at least one gene. The *FHIT*, *FANCF *and *RUNX3 *gene promoters were frequently methylated. Methylation status was correlated with histologic characteristics. We also found evidence that promoter methylation inactivates gene expression in GCTs and exposure to methylation and/or histone deacetylase (HDAC)-inhibiting agents reactivate the gene expression.

## Results

**Figure 1:**
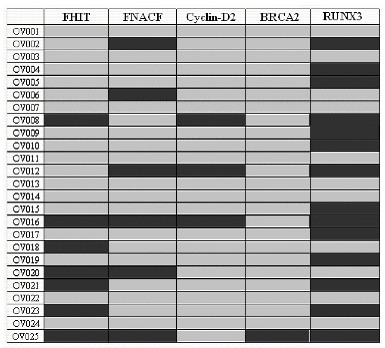
Methylated profile of GCTs of ovarian origin in 25 patients for FHIT, FANCF, cyclin-D2 and BRCa2 genes. Results were scored as methylated (dark boxes) or unmethylated (light grey boxes).

**Figure 2:**
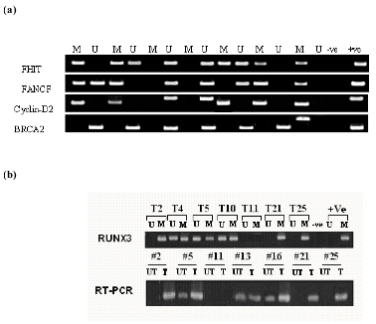
***(a) ***Representative examples of MSP of *FHIT*, *FANCF*, *Cyclin*-*D2 *and *BRCA2 *genes in cell lines and tumor samples; -ve, negative control (water blank PCR mixture without template); +ve, positive control (normal lymphocyte DNA treated with *Sss*I methyl transferase). The unmethylated form of *p16 *was amplified as a control to check DNA integrity. **(b) **Representative examples of MSP with RUNX3 gene and its expression before and after treatment with 5'-AZA-dC in different ovarian tissue (both normal and cancerous) using RT-PCR. UT: untreated; T: treated with 5'-AZA-dC; T2–T25: tumour samples.

We examined the hypermethylation status of a panel of 5 normally unmethylated tumor suppressor or cancer genes: *FHIT*, *FANCF*, *Cyclin-D2*, *BRCA2 *and *RUNX3 *in 25 ovarian GCTs and ovarian cell line DNAs using the MSP assay (Fig. 1). The frequency of promoter hypermethylation of the tumor suppressor gene loci included in the panel was *FHIT 2*8%, *FANCF 24*%, *Cyclin*-*D2 *12%, *BRCA2 *4%, and *RUNX3 *56% of the 25 tumours (Table 1). DNA methylation and mRNA expression results in 5 ovarian cell lines are shown in Table 2. Fig. 2a and 2b shows representative examples of MSP of each gene.

**Table 1:** DNA methylation and mRNA expression in ovarian cancer cell lines

**Cell line**	**MSP**					**mRNA expression**			
	FHIT	FANCF	Cyclin-D2	BRCA2	RUNX3	FHIT	FANCF	Cyclin-D2	RUNX3
TOV-21G	M	M/U	M	U	U	+	-	+	+
C13*	M/U	M	M	U	M	-	+	-	-
OAW42	U	M/U	U	U	U	+	+	+	+
OAW28	U	U	U	U	U	+	+	+	+
OV-90	M/U	U	M	U	U	+	+	-	+
KGN	M	U	U	U	M	+	+	+	-

**Table 2:** Association of grade classification (IA, IB and IC) and promoter methylation of different genes in granulosa cell tumours of ovarian origin

**Genes**	**Methylation Status**	**Grade classification**			p-value*	**p-value**^†^	p-value**	**p-value**^‡^	**p-value**^#^
		**IA**	**IB**	**IC**					
FHIT	Methylated	3	1	3	1	0.0526	0.5856	0.2352	0.041^#^
	Unmethylated	8	9	1					
FANCF	Methylated	2	1	3	0.6609	0.0312^†^	1	0.0769	0.041^#^
	Unmethylated	9	9	1					
Cyclin-D2	Methylated	0	0	3	0.23	0.0017^†^	-	0.0088^‡^	0.011^#^
	Unmethylated	11	10	1					
BRCA2	Methylated	0	0	1	1	0.16	-	0.2667	0.2857
	Unmethylated	11	10	3					
RUNX3	Methylated	7	3	4	0.6887	0.1052	0.1984	0.5165	0.0699
	Unmethylated	4	7	0					

*Fisher's exact test. IA type vs. IB and IC type; †Fisher's exact test. IA and IB type vs. IC type GCTs of ovarian origin;

**Fisher's exact test. IA type vs. IB; ‡Fisher's exact test. IA type vs. IC type; #Fisher's exact test. IB type vs. IC type GCTs of ovarian origin

Hypermethylation was observed in all of the histological stages of cancer examined and in patients of all ages except *BRCA2 *which is seen in only one patient with stage IC. Eighteen tumors showed methylation of at least 1 gene, and 7 tumors showed no methylation of any of the 5 genes. A total of 28% of these tumors had one gene, 16% two genes, 8% three genes and 8% four genes hypermethylated (Fig 1). No methylation was observed in 15 normal ovarian tissue DNAs and 50 lymphocyte DNAs from females. Using statistical analysis, we examined methylation with regard to these cancer patient clinicopathological parameters of age and stage. None of these patients have any smoking history. *FHIT *methylation was only found in all three stages of the tumors but was significantly more pronounced in IC (P = 0.041 vs IB). Hypermethylation of *FANCF *was significantly more frequent in stage IC (P = 0.0312 when compared with IA and IB collectively; P = 0.041 vs IB). *Cyclin*-*D2 *methylation was found only in patients with IC type of cancer (*P *= 0.0017 vs. IA+ IB; P = 0.0088 vs. IA and P = 0.011 vs. IB; Table 1). *BRCA2 *methylation was found only in one patient with stage IC only cancer. *RUNX3 *methylation was found in all the three stages of these tumours. However, all the patients with stage IC showed methylation to associate with high stage but not at a statistically significant level. *FANCF *and *Cyclin-D2 *methylation was found to be more pronounced in older than younger patients. However, we are unable to amplify the modified DNA for either of the PCR products.

Methylation is known to inactivate the tumour suppressor genes. To test the hypothesis, we examined the expression of all these genes except *BRCA2 *by RT-PCR after cellular exposure to 5-Aza-2'-deoxycytidine (DAC). No *FANCF *mRNA in TOV-21G, Cyclin-D2 in C13* and OV-90, and *RUNX3 *in C13* and KGN was detected in untreated cell lines (Table 2). However, DAC treatment for 120 hours induced an increase in the detectable level of mRNA expression in these cell lines and the tumors samples that initially lacked the expression. We were unable to detect expression of RUNX3 gene in patient 11 that also shows no PCR products with either methylated or unmethylated PCR primers. Further studies involving this patient with markers specific for RUNX3 revealed the loss of heterozygosity (LOH) which may be responsible for the loss of expression (Data not given).

## Discussion

Cancer cells especially of ovarian origin keep on accumulating genetic changes that allow them to evade various chemotherapeutic drugs and hence become increasingly dangerous. We try to answer some of the questions by exploring the role of methylation mediated gene silencing in five tumour suppressor gene in the present study. We asked a question whether hypermethylation of *FHIT*, *FANCF*, *cyclin-D2*, *BRCA2 *and *RUNX3 *resulted in the loss of gene expression. We performed reverse transcription-PCR to test the expression of all these genes on RNA extracted from tumour samples. With few exceptions, expression of mRNA correlated well with promoter hypermethylation of these genes. This could be partly due to complex process that controls gene expression in which the chromatin conformation, cofactors availability, repressor process and enhancer molecules all play a part. The present study shows that methylation is one of the important determinants, because in majority of the cases, expression of these genes in these tumours correlates with hypermethylation of the promoter sequences. We found a link between aberrant methylation of genes investigated and the clinicopathological features of stage IC, even though the sample size was small. One has to bear in mind that GCTs of ovarian origin constitute less than 5% of the total ovarian cancers. Similar reports have been reported for the *FHIT *methylation in highly malignant osteosarcomatous [21]. Taken together, our results demonstrate that promoter aberrant methylation of *FHIT *is an important mechanism for inactivation of this tumor suppressor gene in many malignancies. Cytogenetic investigation in these tumours revealed that chromosome 3 and 11 are found to carry deletions and reciprocal translocations along with the trisomy 14 and monosomy 22. It could be important because some of the tumour suppressor genes (*FHIT*, *RASSF1A*, *RAR-β *and *FANCF*) studied extensively in these tumours are in fact localised on these chromosomes.

FANC genes are essential in DNA repair pathways and recently, it has been shown that promoter hypermethylation of *FANCF *gene disrupts the FA-BRCA pathway, resulting in cisplastin resistance. We found *FANCF *promoter hypermethylation in 24% of the tumours. This is in line with the previous findings in squamous cell carcinomas of lung and oral cavity and, in cervical and ovarian cancer [22-25]. To test whether other epigenetic mechanisms such as partial methylation and histone deacetylation play a role, we examined the expression of all these genes except *BRCA2 *after treatment with DAC. DNA hypermethylation-mediated gene silencing is closely associated with histone modifications such as methyl-H3-K9. In this regard, DNA-demethylating agents 5'-aza-2'deoxycytidine (DAC) and trichostatin (TSA) reactivates expression of epigenetically silenced genes [26]. Although DNA hypermethylation is essential to maintain repressive state of histone code, histone modifications precede DNA hypermethylation in silencing specific genes [27,28]. In the present study, reactivation and/or increased expression of *FHIT*, *cyclin-D2 *and *RUNX3 *in C13*, *FANCF *in TOV-21G and *cyclin-D2 *in OV-90 cell line after exposure to DAC in the absence of promoter methylation suggests that key histone modifications, either by direct or indirect involvement of promoter methylation, also play a role in down-regulating *FANCF *gene expression in this cancer type. It is therefore assumed that *FANCF *silencing might be considered a candidate-mechanism underlying the state of genomic instability may be considered to be a rate-limiting in the origin of GCTs of ovarian origin. *FANCF *gene silencing has been shown to revert in vitro as a result of demethylation in some ovarian cell lines as seen in the present study also [23]. These reversible changes in the methylation status further complicate the tumour assessment that shows the contribution of *FANCF *methylation. The present finding along with the results from other cancer types suggests the presence of genomic instability due to the methylation mediated *FANCF *gene silencing. *Cyclin D2 *gene promoter is hypermethylated in 12% of GCTs, a number that is consistent with previous findings in other cancers [14,29,30]. We noted a trend that *Cyclin D2 *methylation is found only in IC type tumors.

The present study showed that hypermethylation of the *RUNX3 *gene promoter frequently occurred in ovarian GCTs. In human gastric cancers it has been shown that promoter hypermethylation and hemizygous deletion of the *RUNX3 *gene correlated with a significant reduction in expression, and the tumorigenicity of cell lines in nude mice was inversely related to their level of *RUNX3 *expression, indicating that *RUNX3 *is a tumor suppressor involved in the development of gastric cancers [17]. The presence of *RUNX3 *CpG island hypermethylation in GCTs, but not in normal ovarian tissue or peripheral blood suggests that *RUNX3 *hypermethylation might be associated with the genesis of this cancer type and this frequent methylation might serve as a biological marker. Loss of expression in RUNX3 gene in patient 11 which also lacks any PCR product with either of the unmethylated and methylated primers indicates that it could be due to the large deletion in the gene. However, studies involving the markers D1S234 and D1S199 showed the loss of heterozygosity which could to some extent explain the loss of expression in this patient. We could not confirm this due to the lack of further sample. However, the sample quality was good as indicated by the presence of band in *GAPDH1 *which was used as an internal control.

The histological examination of granulosa cells revealed that these are small, usually round to polygonal, but may be spindle-shaped with scanty amphophilic cytoplasm, containing only occasional small lipid droplets, and having indistinct cell borders. Granulosa cells regularly express inhibin and contain vimentin and smooth muscle actin intermediate filaments and, less commonly, cytokeratins. However, during their malignant transformation, theses cells exhibit one or more of so-called 'microfollicular', 'macrofollicular', 'trabecular' or 'insular' patterns. The microfollicular variant is characterized by multiple small rounded spaces formed by cystic degeneration in small aggregates of granulosa cells and often fragments of nuclear debris or pyknotic nuclei. These spaces, known as Call-Exner bodies, are found in only 30–50% of tumors. The granulosa cell nuclei are oriented somewhat radially around these structures. The nuclei are typically bland and often grooved in granulosa cell tumors and have few mitoses, but show variation in size and shape, and marked hyperchromatism.

There exist two different pathways that can contribute to the development of cancers; genome-wide hypomethylation may lead to the loss of chromosomes (as seen in these tumours) leading to chromosomal instability, whereas promoter methylation in tumour suppressor genes, which are responsible for gene silencing, can lead to the development of cancers in somatic cells. Therefore, the balance in DNA methylation is very important, and alteration in these may be protective in one pathway but deleterious in the other.

## Conclusions

In view of the high mortality rates associated with ovarian cancer, a better understanding of the molecular mechanisms underlying tumor progression in the disease could reveal novel pathways of high clinical relevance. It can therefore be concluded that promoter hypermethylation of these genes, and loss of expression in ovarian cancers (GCTs) is relatively common and this may also be useful as a tumour marker for early diagnosis and subsequent disease monitoring. Hence, these epigenetic signatures could play a decisive role in designing treatment options for this category of ovarian cancer. These results may also have implications in better understanding the underlying epigenetic mechanisms in GCT development thus can provide prognostic indicators, and identify important gene targets for treatment of this type of cancer in females.

## Methods

### Study Population

The subjects were 25 patients affected with GCTs of ovarian origin without a positive family history. The study was approved by the Health Research Ethics Board of the Faculty of Natural Sciences. All of them were untreated at the time of study. Fifty normal individuals ranging in age from 20–60 years were studied simultaneously under similar experimental conditions. The tumor grading is as follows: 11 FIGO stage IA, 10 FIGO stage IB and 4 FIGO stage IC. All these diagnoses were reviewed by a gynaecologic pathologist and the tumor were assessed using standard criteria. An informed consent was taken from all the subjects prior the study. Fresh cancer tissue specimens were received from all the patient and these were cultured as per standard protocol. A part of the fresh tissue was used to isolate DNA and RNA for further analysis where as the cultured cells were used for the 5'-Aza-2'-deoxycytidine experiments. Microscopically, GCTs are composed of granulosa cells, theca cells, and fibroblasts in varying amounts and combinations. The term granulosa-theca cell tumor had been applied to all tumors in which both cell types were identified, regardless of the amounts present.

### Cell Lines and DNA Isolation

Five human ovarian cancer cell lines (TOV21G, C13*, OAW28, OAW42, OV90 and KGN) from the American Type Culture Collection and European Collection of Cell Culture were maintained in RPMI 1640 supplemented with 10% fetal bovine serum (Hyclone, Logan, UT) and were grown at 37°C in 5% CO_2 _[31]. DNA was isolated from cultured cells using QIAamp DNA Mini kit (Qiagen Inc.) and quantified. LOH studies were performed using markers D1S199 and D1S234 specific for locus 1p36.11.

### Methylation Status by Methylation-Specific PCR (MS-PCR)

DNA methylation patterns in CpG islands of tumor suppressor genes FHIT, FNACF and BRCA2 were determined by chemical modification with sodium bisulphite as described previously [32]. Briefly, 1 μg DNA l was denatured by NaOH (50 μl, final concentration, 0.2 M) for 10 min at 37°C. 1 μg of salmon sperm DNA (Sigma) was added as carrier before modification. Freshly prepared 30 μl of hydroquinone (10 mM, Sigma) and 520 μl of sodium bisulfite (3 M, pH 5.0, Sigma) were mixed and samples were incubated under mineral oil at 55°C for 16 hr. The DNA samples were desalted through Wizard columns (Promega, Madison, WI), desulfonated by NaOH (final concentration, 0.3 M) for 5 min at room temperature, followed by ethanol precipitation. DNA was resuspended in water and used immediately or stored at -20°C. 50 μl of bisulphite modified DNA was used for each MSP. Following primer pairs for *FHIT *gene, methylated CpG site, forward 5'-ttggggcgcgggtttgggtttttacgc-3' and reverse 5'-cgtaaacgacgccgaccccacta-3', unmethylated CpG site, forward 5'-ttggggtgtgggtttgggtttttatg-3', and reverse 5'-cataaacaacaccaaccccacta-3'; 189–262 bp relative to transcription start site, *FANCF *gene, methylated CpG site, forward 5'-tttttgcgtttgttggagaatcgggttttc-3' and reverse 5'-atacaccgcaaaccgccgacgaacaaaacg-3', unmethylated CpG site, forward 5'-tttttgtgtttgttggagaattgggttttt-3' and reverse 5'-atacaccacaaaccaccaacaaacaaaaca-3'; the primers corresponds to the position +280 to +432, *BRCA2 *gene, methylated CpG site, forward 5'-gacggttgggatgtttgataagg-3' and reverse 5'-aatctatcccctcacgcttctcc-3', unmethylated CpG site, forward 5'-agggtggtttgggatttttaagg-3' and reverse 5'-tcacacttctcccaacaacaacc-3'; these primers are 135 and 211 bp upstream of transcription start site and *cyclin-D2 *gene, methylated, forward 5'-tacgtgttagggtcgatcg-3' (-1427 to -1409) and reverse 5'-cgaaatatctacgctaaacg-3' (-1152 to -1171) and unmethylated, forward 5'-gttatgttatgtttgttgtatg-3' (-1616 to -1594) and reverse 5'-taaaatccaccaacacaatca-3' (-1394 to -1414). Each PCR reaction generated 74 bp products both with methylated and unmethylated primers for *FHIT*, 153 bp for *FANCF *both with methylated and unmethylated primers, 337 and 250 bp product with *BRCA2 *primers specific for methylated and unmethylated primers and 276 and 222 bp product for *cyclin-D2 *primers specific for methylated and unmethylated PCR reactions respectively. For the PCR reaction of 25 μl, 50 ng sodium bisulfite treated DNA was added to reaction buffer containing 0.2 mM dNTP, 16.6 mM (NH4)_2_SO4, 67 mM Tris pH 8.8, 10 mM β-mercaptoethanol, 1.5 mM MgCl2, 10 pmol of forward and reverse primers specific to the methylated and methylated DNA sequences and 1.25 units of AmpliTaq Gold (PE Biosystems, Foster City, CA, USA). The PCR reactions were cycled in a GeneAmp 9600 thermal cycler (Applied Biosystems) under the following conditions: preheat at 94°C for 3 min. followed by 40 cycles (94°C for 40 sec, 65°C for 40 sec, for *FHIT*, 4 cycles of 65°C and 36 cycles of 55°C for 60 seconds for *FANCF *and 62°C or 56°C for 40 seconds in methylated or unmethylated BRCA2 gene, 72°C for 45 sec and a final extension at 72°C for 7 min. The PCR conditions for cyclin-D2 are as follows: 1 cycle of 95°C for 5 min and 35 cycles at 95°C for 30 s, 55°C for 30 s, and 72°C for 45 s; and 1 cycle of 72°C for 5 min. In addition to these genes we also investigated the *RUNX3 *gene (Accession no AL023096) methylation status using the primers: forward, 5'-ataatagcggtcgttagggcgtcg-3', and reverse, 5'-gcttctactttcccgcttctcgcg-3' (64917–65031; 115 bp), for methylated DNA of *RUNX3*; and forward, 5'-ataatagtggttgttagggtgttg-3', and reverse, 5'-acttctactttcccacttctcaca-3' (64917–65031; 115 bp), for unmethylated DNA of *RUNX3 *with annealing temperature of 55°C for 20 seconds. For each PCR set, DNA isolated from normal peripheral lymphocytes of healthy individuals served as a negative methylation control. Human placental DNA was treated in vitro with SssI methyltransferase (NEB, Beverly, USA) to create completely methylated DNA at all CpG-rich regions and served as positive methylation control. Methylation-specific PCR products were analysed on 3% agarose gel electrophoresis with ethidium bromide staining. A positive control and a negative control were included in each amplification reaction.

### *FHIT*, *FANCF*, *Cyclin-D2 *and *RUNX3 *Expression by RT-PCR

*FHIT*, *FNACF *and *cyclin-D2 *mRNA levels in cancer patients were compared to their expression in normal cells using semi-quantitative reverse transcriptase polymerase chain reaction. In brief, cDNA was synthesized using AMV reverse transcriptase (Promega, Mannheim, Germany) and amplified in duplex reactions in a total volume of 25 μl containing 150 μM of each dNTP, 1.5 mM MgCl2, 10 pmol of each primer pair and 1.0 units of Taq polymerase. After initial denaturation at 95°C (94°C) for 5 min, 30 cycles of 30 sec (45 sec) at 95°C (94°C), 30 sec (50 sec) at 58°C (55°C) and 45 sec (50 sec) at 72°C were performed followed by a final 10 min elongation step at 72°C. The values in the brackets correspond to *FANCF*. The following primers were used to amplify *FHIT*: forward 5'-gctcttgtgaataggaaacc-3' and reverse 5'-tcactggttgaagaatacagg-3' which yields 532 bp product spanning within exon 5 to exon 10, *FANCF*: forward 5'-ttcggaagtctttgctgcct-3' and reverse 5'-agtaataacacacgattgcc-3' which yields 413 bp product spanning from +733 to +1144. RT-PCR was performed for *Cyclin D2 *using the primers 5'-catggagctgctgtgccacg-3' (forward) and 5'-ccgacctacctccagcatcc-3' (reverse) with PCR conditions being: 1 cycle of 94°C for 3 min and 35 cycles at 94°C for 20 s, 55°C for 30 s, 72°C for 45 s followed by 72°C for 5 min. RT-PCR was also carried out for *RUNX3 *gene using the primers: forward 5'-aggcattgcgcagctcagcggagta-3' and reverse 5'-tctgctccgtgctgccctcgcactg-3' (152 bp). *GAPDH1 *was used as internal control. Each reaction was performed in triplicate. PCR products were electrophoresed on 2% agarose gels and quantified using densitometer (Molecular Dynamics). Fold increases in expression in cancer cells were calculated with respect to the levels of the transcripts in normal cells.

### 5-Aza-2'-deoxycytidine and n-butyrate treatment

Cell lines (TOV21G, C13*, OAW28, OAW42 and OV90), normal ovarian cells and tumour cells of ovarian GCTs were treated with demethylating agent 5-Aza-2' deoxycytidine (Sigma) for five days at a concentration of 2.5 μM, HDAC-inhibiting agent trichostatin (TSA) at a final concentration of 5 μM for the last 24 hours or a combination of both. Total RNA isolated from treated, untreated cell lines and cell of the cancerous tissue was reverse transcribed using random primers and the Pro-STAR first strand RT-PCR kit (Stratagene, La Jolla, CA). A semi-quantitative analysis of gene expression was performed in replicate experiments using 30 cycles of RT-PCR as described above.

## Competing Interests

The authors declare that they have no competing interests.

## Authors' Contributions

VSD for executing the MSP and RT-PCR experiments; completing manuscript; MS, for carrying out cell culturing; and SAH conceived and coordinated the study. All authors read and approved the final manuscript.

**Figure 3:**
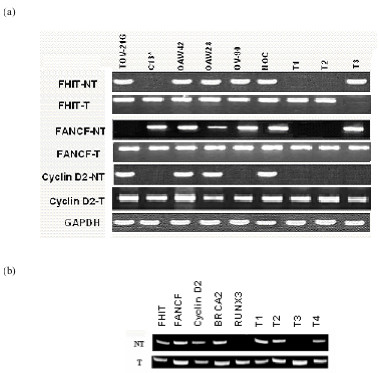
**(a). **Expression of FHIT, FANCF and Cyclin D2 genes before and after treatment with 5'-AZA-dC in different cell lines and ovarian tissue (both normal and cancerous) using RT-PCR. NT: no treatment; T: treatment with 5'-AZA-dC; T1–T3: tumour samples; NOV: normal ovarian cells. Co-amplified product of GAPDH served as an internal control. **(b) **Results obtained with another cell line KGN and tumours before and after treatment with 5'-AZA-dC.
